# Gender Differences in Traditional Chinese Medicine Use among Adults in Taiwan

**DOI:** 10.1371/journal.pone.0032540

**Published:** 2012-04-23

**Authors:** Chun-Chuan Shih, Chien-Chang Liao, Yi-Chang Su, Chin-Chuan Tsai, Jaung-Geng Lin

**Affiliations:** 1 The School of Chinese Medicine for Post-Baccalaureate, I-Shou University, Kaohsiung County, Taiwan; 2 Graduate Institute of Chinese Medicine, College of Chinese Medicine, China Medical University, Taichung, Taiwan; 3 Taipei Chinese Medical Association, Taipei, Taiwan; 4 Department of Anesthesiology, Taipei Medical University Hospital, Taipei, Taiwan; 5 School of Medicine, Taipei Medical University, Taipei, Taiwan; 6 Center for Health Research, Taipei Medical University Hospital, Taipei, Taiwan; 7 Management Office for Health Data, China Medical University Hospital, Taichung, Taiwan; Johns Hopkins Bloomberg School of Public Health, United States of America

## Abstract

**Objectives:**

The increasing use of complementary, alternative medicine (CAM) and traditional Chinese medicine (TCM) has attracted attention. We report on the gender difference in TCM use among the general population in Taiwan in a population-based, cross-sectional study.

**Methods:**

We collected data on socio-demographic factors, lifestyle and health behavior from the 2001 Taiwan National Health Interview Survey. The medical records of interviewees aged 20–69 years were obtained from National Health Insurance claims data with informed consent. The prevalence of TCM use and the average frequency of TCM use were compared between women and men.

**Results:**

Among 14,064 eligible participants, the one-year prevalence of TCM use for women and men was 31.8% and 22.4%, respectively. Compared with men, women had a higher average TCM use frequency (1.55 visits vs. 1.04 visits, p<0.001). This significant difference remained evident after excluding gender-specific diseases (1.43 visits vs. 1.03 visits, p<0.001). The average TCM use frequency was significantly higher in women than in men across all age groups. TCM use correlates differed for women and men. Marital status (odds ratio [OR] = 1.55, 95% confidence interval [CI] = 1.30–1.85), family income and unhealthy lifestyle (OR = 1.50, 95% CI = 1.30–1.74) were factors associated with TCM use in men but not in women.

**Conclusions:**

In Taiwan, women used more TCM services than men and the gender differences in the TCM use profile persisted across age groups.

## Introduction

The increasing use of complementary and alternative medicine (CAM) in recent decades has been documented in many studies in both Asian and Western countries [1]–[5]. In the United Kingdom an estimated 46% for lifetime use of one or more CAM therapies has been reported in a population-based survey [3]. In Israel, the percentage of the Jewish population aged 45–75 years who consulted CAM providers during the previous year increased from 6% in 1993 to 10% in 2000 [4]. A high prevalence of CAM use has also been observed in Scandinavian countries [5]. This interesting phenomenon has attracted physicians' attention. Many physicians recognize that their patients are interested in CAM therapies and some physicians are interested in learning more about CAM [6], [7].

The utilization of traditional Chinese medicine (TCM) is common in Taiwan, Asian countries and immigrant Asians in western countries [8]–[19]. It was estimated that the one-month, one-year, and six-year utilizations of TCM services in Taiwan were 10.4%, 28.4%, and 62.5%, respectively [12], [20]. This pattern of medical pluralism has been investigated in Taiwan [11]. The growing use of TCM in western countries has also been reported [21]–[23]. Previous reports have focused on the prevalence of TCM use and associated factors. Women were found to have higher TCM use than men [12], [20], [24]–[27]. However, few studies investigated the nature of this gender difference in TCM utilization.

Using the National Health Interview Survey (NHIS) and data from the National Health Insurance (NHI) system, this study reports on factors associated with TCM use among adults aged 18 years and older in Taiwan and focuses particularly on the gender difference in TCM usage. The objective of this study is to clarify the gender difference in TCM utilization in Taiwan. This study also provides women’s healthcare information on CAM for health professionals.

## Materials and Methods

### Ethics Statement

This study is based in part on data from the 2001 NHIS approved by the Bureau of Health Promotion, Department of Health and National Health Research Institutes. The Bureau of Health Promotion, Department of Health obtained written informed consent from eligible NHIS participants. The data were analyzed anonymously and all clinical investigations were conducted according to the principles expressed in the Declaration of Helsinki.

### Study Design

The 2001 NHIS was a cross-sectional household survey with a representative sample of the non-institutionalized population. Interviewees were all residents of Taiwan, and interviewers completed the interviews in participants’ homes. All interviewees were selected from the household census in Taiwan. In addition to personal and socio-demographic information, a face-to-face interview survey also collected information regarding individuals’ personal health factors (e.g., disease history and lifestyle), environmental health factors (e.g., exposure to second-hand smoke and home safety), and medical service use (e.g., patterns of medical service use and insurance). Interviews for the population-based survey were completed for 22,121 individuals in 5,798 households at the end of 2001 [28]. About 87% (n = 19,143) of the interviewees gave informed consent for us to link their information with the NHI claims data to retrieve information on medical service use in 2001, including TCM visits. Among the 19,143 participants who agreed to the linking, 14,064 (73.5%) adults between 20 and 69 years old were included in our analysis. Adults in this age group were selected for two reasons: we assumed people in this age group usually made independent decisions about their health care-seeking behaviors and we observed that gender differences were not significant for people aged 70 or older.

Both NHIS data and NHI claims data from 2001 were used in this study. With the written informed consent of eligible NHIS participants that allowed the linking of their NHI data, the 2001 NHIS data were linked to the 2001 NHI claims data. Information about prevalence and frequency of TCM use was drawn from the 2001 NHI claims data. The data were limited to visits to ambulatory TCM medical facilities contracted with the Bureau of National Health Insurance in Taiwan, including 2 public TCM hospitals, 42 private TCM hospitals and 2,544 private TCM clinics in 2001 [29]. Socio-demographic data (including age, gender, education, occupation, family income, ethnicity, religion, and marital status), use of folk therapy (FT), regular health checkups and unhealthy lifestyle (including smoking, alcohol use, and betel nut chewing) were derived from the NHIS data. The FT utilization question in the questionnaire is as follows: In the past one month, have you undergone any therapy such as Gua Sha (skin scraping), Tuina (massage and kneading), Baguan (vacuum bottle therapy), bone setting, spine alignment, Qigong, divination, written charms, sharman, talisman, incense ash or other FT to ease physical discomfort in some non-medical places? (Legal traditional Chinese medicine and western medicine excluded).

**Table 1:** Characteristics of study participants among men and women.

		Use of TCM			
		Female,n (%)		Male,n (%)	
Age, years	20–29	1798	(33.4)	1751	(21.9)
	30–39	1742	(32.8)	1736	(23.8)
	40–49	1720	(32.2)	1650	(23.2)
	50–59	1093	(32.0)	1012	(20.8)
	60–69	820	(25.4)	742	(20.4)
Education, years	0	711	(24.5)	192	(14.6)
	1–9	2554	(31.8)	2409	(21.9)
	10–12	2166	(32.2)	2210	(22.5)
	≥13	1736	(34.4)	2076	(23.5)
Occupation	Unemployed	872	(30.2)	1703	(20.7)
	Homemaker	2355	(29.3)	12	(25.0)
	Skilled, Unskilled	3207	(33.8)	3790	(22.3)
	Profession	712	(34.0)	1353	(24.5)
Family income, NTDs	<30,000	1304	(29.2)	1162	(18.7)
	30,000–49,999	1584	(31.8)	1578	(23.6)
	50,000–99,999	2943	(33.0)	2831	(24.1)
	≥100,000	1266	(31.9)	1263	(20.7)
Religion	No religion	1904	(29.3)	1857	(21.3)
	Traditional religions	3346	(32.7)	3393	(22.4)
	Buddhist	1493	(35.2)	1259	(24.5)
	Others^a^	417	(24.5)	366	(19.7)
Marital status	Married	4933	(31.1)	4570	(23.9)
	Other status	2240	(33.5)	2321	(19.3)
Indigenous		208	(19.2)	189	(18.5)
Use of folk therapy		518	(44.4)	307	(36.5)
Have regular health checkup		1089	(34.6)	1114	(24.8)
Number of unhealthy lifestyles	None	6084	(32.0)	1905	(25.8)
	One	829	(32.6)	1831	(23.0)
	Two or three	256	(25.4)	3153	(19.9)
Urbanization	Low	2442	(28.4)	2469	(19.2)
	Moderate	3232	(32.1)	3036	(22.8)
	High	1499	(37.0)	1386	(27.1)
Density of TCM physicians	Low	2222	(31.8)	2274	(20.3)
	Moderate	1468	(32.6)	1365	(24.4)
	High	3483	(31.6)	3252	(22.9)

TCM, traditional Chinese medicine; NTDs, New Taiwan Dollars.

a Others: Including Protestantism, Catholicism or Islam.

**Table 2:** The average frequency of TCM visits per year for women and men by age groups.

	Average frequency of TCM visits per year						
	All diseases				Diseases excluding gender-specific diseases		
Age group	Women	Men			Women	Men	
All ages	1.55	1.04	p<0.001		1.43	1.03	p<0.001
20–29	1.46	0.84	p<0.001		1.27	0.83	p<0.001
30–39	1.63	0.99	p<0.001		1.44	0.98	p<0.001
40–49	1.60	1.05	p<0.001		1.50	1.05	p<0.001
50–59	1.71	1.31	p<0.001		1.66	1.30	p<0.001
60–69	1.31	1.23	p<0.05		1.30	1.20	p<0.05

TCM, traditional Chinese medicine. All p-values were estimated using Wilcoxon rank sum tests.

### Definitions and Measures

#### TCM

TCM includes herbal medicines, acupuncture, moxibustion, bone reduction, traditional trauma treatment, traditional dislocation treatment, traditional fracture treatment, Tuina, Baguan, and other therapies. TCM practitioners are registered TCM physicians and practice in a hospital or clinic. TCM in Taiwan is legal, and TCM physicians can advertise the medical benefits of TCM according to medical laws in Taiwan [12].

#### Folk therapy (FT)

FT use is defined as utilization of FT within the past month. The types of FT studied include Gua Sha (skin scraping), Tuina (massage and kneading), Baguan (vacuum bottle therapy), bone setting, spine alignment, Qigong, divination, written charms, shaman consultation, talismans, incense ash, and other related therapies. The difference between TCM and FT lies in their legality. Legislation from the Department of Health in Taiwan has declared that FT practitioners cannot claim any medical benefits from FT. FT practitioners do not have certifications or legal licenses and they do not work in clinical settings in Taiwan [12].

#### Urbanization

There are 359 townships and city districts in Taiwan. We calculated the population density (persons/km^2^) by dividing the population (persons) by the area (km^2^) for each of these administrative units according to the statistics from Minister of Interior, Taiwan. The first, second, and third tertiles were considered areas of low, moderate and high urbanization, respectively [12], .

#### Density of TCM physicians

We calculated the density of TCM physicians (TCM physicians/10,000 persons) by using the number of TCM physicians per 10,000 persons for each of the administrative units. The first, second, and third tertiles were considered areas of low, moderate, and high TCM physician density, respectively [12].

#### Unhealthy lifestyle

A high prevalence of cigarette smoking, alcohol drinking, and betel quid chewing has been found in Taiwan [31]. People with the habit of smoking tobacco (included some times of smoking in lifetime; or less than 5 package in lifetime; or more than 5 package in lifetime), drinking alcohol (current drinkers included any types of alcohol drinks) and/or chewing areca were considered to have unhealthy lifestyles that are associated with cancer and other diseases [11], [12].

**Table 3:** Factors associated with TCM use among women and men in multiple logistic models.

Factors		Use of TCM			
		Women, OR (95% CI)		Men, OR (95% CI)	
Age, years	20–29	1.28	(0.97–1.67)	1.37	(1.02–1.83)
	30–39	1.30	(1.00–1.68)	1.25	(0.96–1.63)
	40–49	1.23	(0.96–1.58)	1.07	(0.83–1.39)
	50–59	1.33	(1.04–1.70)	0.91	(0.70–1.19)
	60–69	1.00	(Reference)	1.00	(Reference)
Education, years	0	1.00	(Reference)	1.00	(Reference)
	1–9	1.20	(0.93–1.56)	1.63	(0.94–2.86)
	10–12	1.18	(0.89–1.57)	1.47	(0.83–2.61)
	≥13	1.28	(0.94–1.73)	1.43	(0.80–2.56)
Occupation	Unemployed	1.00	(Reference)	1.00	(Reference)
	Homemaker	0.93	(0.76–1.14)	1.00	(0.21–4.85)
	Skilled, Unskilled	1.09	(0.90–1.31)	0.94	(0.80–1.11)
	Profession	1.01	(0.79–1.28)	1.01	(0.82–1.24)
Family income, NTDs	<30,000	1.00	(0.83–1.21)	1.04	(0.83–1.30)
	30,000–49,999	1.07	(0.90–1.27)	1.29	(1.06–1.56)
	50,000–99,999	1.07	(0.93–1.25)	1.26	(1.06–1.49)
	≥100,000	1.00	(Reference)	1.00	(Reference)
Religion	No religion	1.08	(0.82–1.44)	1.11	(0.78–1.58)
	Traditional religions	1.28	(0.97–1.69)	1.18	(0.84–1.67)
	Buddhist	1.40	(1.05–1.87)	1.33	(0.93–1.90)
	Others	1.00	(Reference)	1.00	(Reference)
Marital Status	Married	0.90	(0.78–1.03)	1.55	(1.30–1.85)
Ethnicity	Non-indigenous	1.65	(1.06–2.57)	0.80	(0.49–1.30)
Use of folk therapy	Yes	1.65	(1.36–2.00)	2.00	(1.56–2.55)
Have regular health checkup	Yes	1.15	(0.99–1.33)	1.18	(1.01–1.39)
Have unhealthy lifestyle	No	1.17	(0.85–1.61)	1.50	(1.30–1.74)
	One	1.20	(0.85–1.69)	1.25	(1.08–1.45)
	Two or three	1.00	(Reference)	1.00	(Reference)
Density of TCM physicians^a^	Low	1.00	(Reference)	1.00	(Reference)
	Moderate	1.27	(1.10–1.48)	1.33	(1.13–1.57)
	High	1.64	(1.38–1.95)	1.69	(1.40–2.04)
Urbanization	Low	1.45	(1.24–1.69)	1.19	(1.01–1.41)
	Moderate	1.20	(1.03–1.41)	1.31	(1.10–1.55)
	High	1.00	(Reference)	1.00	(Reference)

a TCM physicians per 10000 residents.

CI, confidence; OR, odds ratio; TCM, traditional Chinese medicine.

### Statistical Analysis

The proportion of TCM use was compared between women and men using Chi-square tests. We used Wilcoxon rank sum tests to analyze the difference in average use frequency between men and women. A multivariate logistic regression analysis was performed to analyze the factors associated with TCM services use for women and men. These covariates included age, education, family income, occupation, urbanization, religion, ethnicity, marital status, use of folk therapy, regular checkups, unhealthy lifestyle and density of TCM physicians. To precisely clarify the gender difference in TCM visits, gender-specific diseases (Table S1 and Table S2) were excluded from the comparisons of the number of visits and treatments sought between men and women. All analyses were performed using Statistical Analysis Software (SAS), version 9.1 (by SAS Institute Inc., Cary, North Carolina, U.S.A.). A two-sided probability value of <0.05 was considered significant.

**Table 4:** Factors associated with TCM use in multivariate logistic regressions.^a^

Factors	Use of TCM	
	OR	(95%CI)
Gender, female vs. male	1.50	(1.34–1.67)
Age, years		
20–29 vs. 60–69	1.22	(1.01–1.48)
30–39 vs. 60–69	1.21	(1.01–1.46)
40–49 vs. 60–69	1.12	(0.94–1.33)
50–59 vs. 60–69	1.10	(0.92–1.32)
Education, years		
1–9 vs. 0	1.26	(1.00–1.58)
10–12 vs. 0	1.19	(0.93–1.51)
≥13 vs. 0	1.24	(0.97–1.60)
Family income, NTDs		
<30,000 vs. ≥100,000	1.00	(0.83–1.21)
30,000–49,999 vs. ≥100,000	1.11	(0.99–1.19)
50,000–99,999 vs. ≥100,000	1.16	(1.02–1.31)
Ethnicity		
Non-indigenous vs. indigenous	1.16	(0.84–1.59)
Religion		
No religion vs. Others	1.08	(0.87–1.34)
Traditional religions vs. Others	1.22	(0.99–1.51)
Buddhist vs. Others	1.36	(1.09–1.69)
Marital Status		
Married vs. others	1.12	(1.01–1.24)
Use of folk therapy		
Yes vs. no	1.78	(1.53–2.08)
Have regular health checkup		
Yes vs. no	1.16	(1.05–1.30)
Have unhealthy lifestyle		
No vs. two or three	1.37	(1.21–1.56)
One vs. two or three	1.27	(1.12–1.45)
Density of TCM physicians^b^		
Moderate vs. low	1.29	(1.16–1.44)
High vs. low	1.66	(1.46–1.88)
Urbanization		
Low vs. high	1.30	(1.16–1.46)
Moderate vs. high	1.25	(1.12–1.40)

CI, confidence; OR, odds ratio; TCM, traditional Chinese medicine.

a Additional adjusted for occupation which is not significant in the model.

b TCM physicians per 10000 residents.

**Table 5:** The distribution of disease categories for which TCM was sought among women and men.

Disease	Proportion (%)			
	All diseases		Excluding gender-specific diseases	
	Women	Men	Women	Men
Diseases of the respiratory system	19.1	18.9	20.6	19.2
Diseases of the musculoskeletal system and connective tissue	18.1	19.4	19.7	19.6
Injury and poisoning	16.5	18.9	17.9	19.1
Specific symptoms, signs and ill-defined conditions	15.0	13.2	16.3	13.3
Diseases of the genitourinary system	11.7	2.7	4.7	1.6
Diseases of the digestive system	10.2	12.4	11.0	12.6
Diseases of the skin and subcutaneous tissue	3.2	3.2	3.4	3.2
Endocrine, nutritional and metabolic diseases and immune disorders	1.5	2.6	1.6	2.6
Diseases of the circulatory system	1.4	3.3	1.5	3.4
Diseases of the nervous system	1.4	2.0	1.5	2.0
Diseases of the sense organs	0.9	1.4	0.9	1.4
Mental disorders	0.4	0.8	0.5	0.8
Infectious and parasitic diseases	0.4	0.8	0.4	0.8
Malignant neoplasia	0.2	0.3	0.1	0.3
Complications of pregnancy, childbirth and the puerperium	0.1	0.0	-	-
Congenital anomalies	0.1	0.0	0.1	0.0
Other neoplasia	0.0	0.2	0.0	0.2

TCM, traditional Chinese medicine.

## Results

Table 1 shows the characteristics of study participants by gender. The prevalence of TCM use among women and men was 31.8% and 22.4%, respectively (p<0.05). Compared with men, women had a higher average frequency of TCM use (1.55 visits per years vs. 1.04 visits per year, p<0.001). This significant difference was present across all age groups and remained evident after excluding the gender-specific diseases (1.43 visits per year vs. 1.03 visits per year, p<0.001) (Table 2). The frequency of TCM use among women and men increased with age in most of the age groups and decreased sharply in the 60–69-year-old group. This ascending trend was even more noticeable after excluding gender-specific diseases. The peak number of TCM visits was in the 50–59-year-old group for both genders, and the lowest number of TCM visits was observed in the 20–29-year-old group, followed by the 60–69-year-old group after excluding gender-specific diseases.

In the multivariate analysis, the following groups were more likely to have visited licensed TCM clinics and practitioners: women aged 30–39 or 50–59 years old who were non-indigenous, were Buddhists, had used FT in the month prior to the interview, lived in areas where the density of TCM practitioners was moderate or high, or lived in areas with low or moderate levels of urbanization. Years of education, occupation, family income, regular health checkups and unhealthy lifestyle were not significantly associated with TCM use. In men, age, family income, marital status, FT use, regular health checkups, and unhealthy lifestyle were associated with TCM use. Men with the following characteristics were more likely to see TCM practitioners: 20–29 years old, average family income less than 100,000 NTDs per month, married, FT users, use of regular health checkups and one or no indicators of unhealthy lifestyle. In addition, a higher density of TCM practitioners and a moderate level of urbanization were also associated with the likelihood of TCM use for men (Table 3).

In the Table 4, females (OR = 1.50, 95% CI = 1.34–1.67), younger people (OR = 1.22, 95% CI = 1.01–1.48), use of FT (OR = 1.78, 95% CI = 1.53–2.08) and having no unhealthy lifestyle (OR = 1.37, 95% CI = 1.21–1.56) were factors associated with TCM use among adults in Taiwan. People living in areas with high density TCM physicians (OR = 1.66, 95% CI = 1.46–1.88) or low-urbanized area (OR = 1.30, 95% CI = 1.16–1.46) were more like to use TCM than the comparison group.

Table 5 presents the distribution of disease categories for which TCM was sought for both women and men. The top five diseases associated with TCM use by women were diseases of the respiratory system (19.1%); diseases of the musculoskeletal system and connective tissue (18.1%); injury and poisoning (16.5%); symptoms, signs and ill-defined conditions (15.0%); and diseases of the genitourinary system (11.7%). The men’s disease profile differed slightly from the women’s, with diseases of the musculoskeletal system and connective tissue (19.4%) being most prevalent, followed by diseases of the respiratory system (18.9%); injury and poisoning (18.9%); symptoms, signs and ill-defined conditions (13.2%); and diseases of the digestive system (12.4%). Compared with women, men were less likely to go to TCM physicians for genitourinary problems (2.7% vs. 11.7%); the disparity was still apparent after excluding gender-specific diseases (1.6% vs. 4.7%).

## Discussion

Using data from this population based nationwide survey we found that women had higher TCM use than men. The factors associated with TCM use were different between women and men. In addition, we investigated the gender difference in TCM use even after excluding gender-specific diseases. One of the strengths of this study is its use of combined NHIS and NHI data, which provided reliable information on medical care usage to minimize recall bias. To the best of our knowledge, this study is the first to exclude gender-specific diseases in a comparison of TCM use between women and men.

There is reasonably consistent evidence that women are somewhat more likely than men to use TCM [32]. MacPherson et al. analyzed 9,408 acupuncture patients in the United Kingdom (UK) and found that 74% were female [23]. Among schizophrenic patients in Hong Kong, females were more likely to use TCM compared with males (OR = 1.32, 95% CI = 1.09–1.62) [13]. Among Korean American elderly, a higher proportion of women visited TCM centres than men in the past 6 months (21.0% vs. 9.3%, p<0.001) [14]. A higher proportion (80%) of TCM was investigated in Singaporean children but the sex difference in TCM use was not reported [17]. In Hong Kong, TCM is used by more women than men (OR = 1.58, 95% CI = 1.28–1.97) [18]. Using the NHI database, Chen et al. found that women had higher TCM use than men [29]. However, the results of that study were not adequately adjusted for socio-demographic factors. Chang et al. did not exclude gender-specific diseases and reported that the odds of using TCM were higher in females than in males [20]. Another population-based survey failed to investigate the gender difference in TCM use [24]. A study documented that women had higher self-reported TCM use than men, but these results may have been limited due to recall bias in medical usage [12]. Among patients with inflammatory bowel disease, women were more likely to use TCM than men [33]. The findings from our study were similar to previous reports that documented the gender difference in the TCM utilization [12]–[14], [18], [20], [23], [29], [34]. Compared with men, women were more likely to use TCM in this study (OR = 1.5). To avoid both recall bias in medical usage and inadequate adjustment for confounding factors, we linked data from the NHIS with the NHI claims database and we found that women had higher TCM use than men even after excluding gender-specific diseases. Unlike previous reports, this study investigated age-specific gender difference in TCM use.

Many studies investigated the higher rate of TCM use in women. [12]–[14], [18], [20], [23], [29], [34] However, limited information was available on differences in the average TCM use frequency between men and women. Chen et al. reported the frequency of TCM use in Taiwan. However, their study did not compare the average TCM use frequency between women and men [29]. In a population-based survey, the average TCM use frequency was 2.26±2.29 visits among people who had used TCM in Taiwan [12]. Our study is the first to report that women had higher TCM use frequency than men in Taiwan; the average number of TCM visits for women and men were 1.55 and 1.04, respectively. We also investigated the gender difference in TCM use frequency across every age stratum.

Our results showed disproportionate TCM use among women for the genitourinary system disease category (11.7% for women vs. 2.7% for men). Even after excluding gender-specific diseases in the analysis, TCM use in this disease category was still higher in women than in men. The female predominance in TCM use observed both in various studies and in medical settings has been attributed to women’s preference for using TCM therapies for their gynecological problems, including infertility, premenstrual syndrome or postmenstrual discomfort. The considerable reduction in the percentage of TCM use among women attributable to genitourinary system-related illnesses after excluding gender-specific diseases suggests that illnesses specific to women’s health accounted for a significant portion of TCM use by women. In terms of health care consumption, women are strong supporters and consumers of TCM services.

In keeping with other reports, [11], [12], [24], [25], [29] we found that younger adults were more likely to use TCM than the elderly. Ethnicity and cultural factors, such as religion and FT use, were identified as factors associated with TCM use, thus suggesting a distinct user profile for women in Chinese society compared with women in western societies. Culture related variables are also important in determining TCM use among the older Chinese immigrants in Canada [16]. In fact, with the exception of highly religious measures (e.g., subduing demons, drawing divinatory lots for medicine or taking incense ash), procedures such as skin scraping, vacuum bottle therapy, bone setting, herbal plasters, massage and osteopathic spinal manipulation were all common treatments applied by TCM practitioners in medical settings. There might be some cultural purposes rather than medical incentives behind these healthcare seeking behaviors that are worthy of further investigation. The higher odds of TCM use in areas with a higher density of TCM practitioners for women seem to support the argument that more resources will engender more medical services consumption. Women living in highly urbanized areas had higher TCM use in this study. In general, urbanization is positively correlated with the density of medical services. People living in highly urbanized areas have more opportunities to access various conventional or unconventional therapies [11], [12], [20].

There are several possible reasons why women had higher TCM use than men. First, women have better knowledge, attitudes and practices for self-care than men. Women are higher users of health care services in general, including CAM and acupuncture, and they are often the primary agents in family health care utilization decisions [21]. It has also been reported that women have more help-seeking behaviors than men [35]. Compared with men, women are more likely to use any form of health care. This tendency might be amplified when considering TCM use [36]. Second, we suggested that women had more opportunities to use TCM for maintaining regular menstruation, [37] promoting health during pregnancy [38]-[41], and treating post-menopausal syndrome in climacteric age [42]. Third, women may use acupuncture treatment to reduce body weight, waist circumference and body mass index to improve their body image [34], [43]. Furthermore, the opportunity cost of accessing medical care is often lower in women than in men. The proportion of women who are homemakers or without a job is higher than in men. As a result, women have more opportunities to use medical care services.

There are some limitations to this study. First, the cross-sectional study design could not provide information about whether TCM use is increasing or decreasing over time or whether the characteristics of TCM users are changing. Second, recall bias in socio-demographic factors and health behaviors may potentially jeopardize the validity of epidemiologic results. Although some researchers have found differences with regard to characteristics between people who gave consent and those who did not [44], we believe this phenomenon did not bias our results significantly because we found that age and gender distributions were comparable between those who consented and those who refused (not shown in the tables). Another limitation is that NHI system coverage more than 95% TCM services are available all over Taiwan. This means that this study may underestimate the utilization of TCM. In addition, the influence of common practices of CAM in the western countries such as heomeopathy, chiropractic or engery therapy on the TCM utilization in Taiwanese people needed to be further evaluated.

In conclusion, we found that women in Taiwan had higher TCM service use than men and the gender difference in the TCM profile use persisted. This study may provide some clinical implications for CAM that are important to health professionals worldwide. More comprehensive data is needed to clarify the causes of the gender difference in TCM use.

## Supporting Information

Table S1 Male-specific diseases according to International Classification of Diseases, Clinical Modification in the Ninth Edition (ICD-9-CM).
(DOC)

Table S2 Female-specific diseases according to International Classification of Diseases, Clinical Modification in the Ninth Edition (ICD-9-CM).
(DOC)

## References

[1] Eisenberg DM, Davis RB, Ettner SL, Appel S, Wilkey S (1998). Trends in alternative medicine use in the United States, 1990–1997: results of a follow-up national survey.. JAMA.

[2] Tindle H, Davis R, Phillips R, Eisenberg D (2005). Trends in use of complementary and alternative medicine by US adults: 1997–2002.. Altern Ther Health Med.

[3] Thomas K, Nicholl J, Coleman P (2001). Use and expenditure on complementary medicine in England: a population based survey.. Complement Ther Med.

[4] Shmueli A, Shuval J (2004). Use of complementary and alternative medicine in Israel: 2000 vs. 1993.. Isr Med Assoc J.

[5] Hanssen B, Grimsgaard S, Launsø L, Fønnebø V, Falkenberg T (2005). Use of complementary and alternative medicine in the Scandinavian countries.. Scand J Prim Health Care.

[6] Davis MP, Darden PM (2003). Use of complementary and alternative medicine by children in the United States.. Arch Pediatr Adolesc Med.

[7] Sikand A, Laken M (1998). Pediatricians’ experience with and attitudes toward complementary/alternative medicine.. Arch Pediatr Adolesc Med.

[8] Barnes PM, Bloom B, Nahin RL (2008). Complementary and Alternative Medicine Use Among Adults and Children: United States, 2007 National Center for Health Statistics.

[9] Buettner C, Kroenke CH, Phillips RS, Davis RB, Eisenberg DM (2006). Correlates of use of different types of complementary and alternative medicine by breast cancer survivors in the nurses’ health study.. Breast Cancer Res Treat.

[10] Buono MD, Urciuoli O, Marietta P, Padoani W, Leo DD (2001). Alternative medicine in a sample of 655 community-dwelling elderly.. J Psychosom Res.

[11] Shih CC, Su YC, Liao CC, Lin JG (2010). Patterns of medical pluralism among adults: results from the 2001 National Health Interview Survey in Taiwan.. BMC Health Serv Res.

[12] Shih CC, Lin JG, Liao CC, Su YC (2009). The utilization of traditional Chinese medicine and associated factors in Taiwan in 2002.. Chin Med J.

[13] Zhang ZJ, Tan QR, Tong Y, Wang XY, Wang HH (2011). An epidemiological study of concomitant use of Chinese medicine and antipsychotics in schizophrenic patients: implication for herb-drug interaction.. PLoS One.

[14] Kim M, Han HR, Kim KB, Duong DN (2002). The use of traditional and Western medicine among Korean American elderly.. J Community Health.

[15] Chen JX, Hu LS (2006). Traditional chinese medicine for the treatment of chronic prostatitis in China: a systematic review and meta-analysis.. J Altern Complement Med.

[16] Lai D, Chappell N (2007). Use of Traditional Chinese Medicine by older Chinese immigrants in Canada.. Fam Pract.

[17] Loh CH (2009). Use of traditional Chinese medicine in Singapore children: perceptions of parents and paediatricians.. Singapore Med J.

[18] Chung V, Wong E, Woo J, Lo SV, Griffiths S (2007). Use of traditional chinese medicine in the Hong Kong special administrative region of China.. J Altern Complement Med.

[19] Wu AP, Burke A, LeBaron S (2007). Use of traditional medicine by immigrant Chinese patients.. Fam Med.

[20] Chang LC, Huang N, Chou YJ, Lee CH, Kao FY (2008). Utilization patterns of Chinese medicine and Western medicine under the National Health Insurance Program in Taiwan, a population-based study from 1997 to 2003.. BMC Health Serv Res.

[21] Upchurch DM, Burke A, Dye C, Chyu L, Kusunoki Y (2008). A sociobehavioral model of acupuncture use, patterns, and satisfaction among women in the United States, 2002.. Women Health Issues.

[22] Burke A, Upchurch DM, Dye C, Chyu L (2006). Acupuncture use in the United States: findings from the National Health Interview Survey.. J Altern Complement Med.

[23] MacPherson H, Sinclair-Lian N, Thomas K (2006). Patients seeking care from acupuncture practitioners in the UK: a national survey.. Complement Ther Med.

[24] Shih SF, Lew-Ting CY, Chang HY, Kuo KN (2008). Insurance covered and non-covered complementary and alternative medicine utilisation among adults in Taiwan.. Soc Sci Med.

[25] Daly M, Tai CJ, Deng CY, Chien LY (2009). Factors associated with utilization of traditional Chinese medicine by white collar foreign workers living in Taiwan.. BMC Health Serv Res.

[26] Chung VCH, Lau CH, Yeoh EK, Griffiths SM (2009). Age, chronic non-communicable disease and choice of traditional Chinese and western medicine outpatient services in a Chinese population.. BMC Health Serv Res.

[27] Pu CY, Lan VM, Lan CF, Lang HC (2008). The determinants of traditional Chinese medicine and acupuncture utilization for cancer patients with simultaneous conventional treatment.. Eur J Cancer Care.

[28] Shih YT, Hung YT, Chang HY, Liu JP, Lin HS (2003). The design, contents, operation and the characteristics of the respondents of the 2001 National Health Interview Survey in Taiwan.. Taiwan J Public Health.

[29] Chen FP, Chen TJ, Kung YY, Chen YC, Chou LF (2007). Use frequency of traditional Chinese medicine in Taiwan.. BMC Health Serv Res.

[30] Liao CC, Li TC, Lin RS, Sung FC (2006). Urban and rural difference in prevalence and incidence of stroke in 2000 in Taiwan.. Taiwan J Public Health.

[31] Liao CC, Wang HY, Lin RS, Hsieh CY, Sung FC (2005). Colorectal and prostate cancer screening practices among men in Taiwan.. Taiwan J Public Health.

[32] Chi CH, Lee JL, Lai JS, Chen CY, Chang SK (1996). The practice of Chinese medicine in Taiwan.. Soc Sci Med.

[33] Chen YC, Chen FP, Chen TJ, Chou LF, Hwang SJ (2008). Patterns of traditional Chinese medicine use in patients with inflammatory bowel disease: a population study in Taiwan.. Hepatogastroenterology.

[34] Hsu CH, Wang CJ, Hwang KC, Lee TY, Chou P (2009). The effect of Auricular acupuncture in obese women: a randomized controlled trial.. J Womens Health.

[35] Oliver MI, Pearson N, Coe N, Gunnell D (2005). Help-seeking behaviour in man and women with common mental health problem: cross-sectional study. Br J Psychiatr.

[36] Bishop FL, Lewith GT (2008). Who uses CAM? A narrative review of demographic characteristics and health: factors associated with CAM use.. Evid Based Complement Alternat Med.

[37] Yeh LLL, Liu JY, Lin KS, Liu YS, Chiou JM (2007). A randomised placebo-controlled trial of a traditional Chinese herbal formula in the treatment of primary dysmenorrhoea.. PLoS ONE 2007;.

[38] Chang PJ, Tseng YC, Chuang CH, Chen YC, Hsieh WS (2010). Use of Sheng-Hua-Tang and health-related quality of life in postpartum women: a population-based cohort study in Taiwan.. Int J Nurs Stud.

[39] Chuang CH, Chang PJ, Hsieh WS, Tsai YJ, Lin SJ (2009). Chinese herbal medicine use in Taiwan during pregnancy and the postpartum period: a population-based cohort study.. Int J Nurs Stud.

[40] Chuang CH, Hsieh WS, Guo YL, Tsai YJ, Chang PJ (2007). Chinese herbal medicines used in pregnancy: a population-based survey in Taiwan.. Pharmacoepidemiol Drug Saf.

[41] Chuang CH, Doyle P, Wang JD, Chang PJ, Lai JN (2006). Herbal medicines used during the first trimester and major congenital malformations: an analysis of data from a pregnancy cohort study.. Drug Saf.

[42] Yang YH, Chen PC, Wang JD, Lee CH, Lai JN (2009). Prescription pattern of traditional Chinese medicine for climacteric women in Taiwan.. Climacteric.

[43] Hsu CH, Hwang KC, Chao CL, Chang HH, Chou P (2005). Electroacupuncture in obese women: a randomized, controlled pilot study.. J Womens Health.

[44] Huang N, Shih SF, Chang HY, Chou YJ (2007). Record linkage research and informed consent: who consents?. BMC Health Serv Res.

