# Supplementary material for: Gender Differences in Traditional Chinese Medicine Use among Adults in Taiwan
Source: PLoS One. 2012 Apr 23;7(4):e32540. doi: 10.1371/journal.pone.0032540 (PMC3335160; doi:10.1371/journal.pone.0032540)
Supplement: Table S1 — Male-specific diseases according to International Classification of Diseases, Clinical Modification in the Ninth Edition (ICD-9-CM). (DOC) [file pone.0032540.s001.doc]

| ICD-9-CM code | Diseases |
| --- | --- |
| 072 | Mumps orchitis |
| 121.0 | Opisthorchiasis |
| 175 | Malignant neoplasm of male breast |
| 185-187 | Malignant neoplasm of prostate; Malignant neoplasm of testis; Malignant neoplasm of penis and other male genital organs |
| 214.4 | Lipoma of spermatic cord |
| 222 | Benign neoplasm of male genital organs |
| 233.4-233.6 | Carcinoma in situ of prostate; Carcinoma in situ of penis; Carcinoma in situ of other and unspecified male genital organs |
| 236.4-236.6 | Neoplasm of uncertain behavior of testis; Neoplasm of uncertain behavior of prostate; Neoplasm of uncertain behavior of other and unspecified male genital organs |
| 257 | Testicular dysfunction |
| 302.74-302.75 | Psychosexual dysfunction with inhibited male orgasm; Psychosexual dysfunction with inhibited premature ejaculation |
| 456.4 | Scrotal varices |
| 600-608 | Diseases of male genital organs |
| 752.5 | Undescended and retractile testicle |
| 792.2 | Nonspecific abnormal findings in semen |
| 878.0-878.3 | Open wound of penis, including traumatic amputation; Open wound of scrotum and testes, including traumatic amputation |
| 939.3 | Foreign body in penis |
|  | |
