# Supplementary material for: Gender Differences in Traditional Chinese Medicine Use among Adults in Taiwan
Source: PLoS One. 2012 Apr 23;7(4):e32540. doi: 10.1371/journal.pone.0032540 (PMC3335160; doi:10.1371/journal.pone.0032540)
Supplement: Table S2 — Female-specific diseases according to International Classification of Diseases, Clinical Modification in the Ninth Edition (ICD-9-CM). (DOC) [file pone.0032540.s002.doc]

| ICD-9-CM code | Diseases |
| --- | --- |
| 023.1 | Brucella abortus |
| 112.1 | Candidiasis of vulva and vagina |
| 174 | Malignant neoplasm of female breast |
| 179-184 | Malignant neoplasm of uterus; Malignant neoplasm of cervix uteri; Malignant neoplasm of placenta; Malignant neoplasm of body of uterus; Malignant neoplasm of ovary and other uterine adnexa; Malignant neoplasm of other and unspecified female genital organs |
| 198.6 | Secondary malignant neoplasm of ovary |
| 218-221 | Uterine leiomyoma; Other benign neoplasm of uterus; Benign neoplasm of ovary; Benign neoplasm of other female genital organs |
| 233.1-233.3 | Carcinoma in situ of cervix uteri; Carcinoma in situ of other and unspecified parts of uterus; Carcinoma in situ of other and unspecified female genital organs |
| 233.1-233.3 | Neoplasm of uncertain behavior of uterus; Neoplasm of uncertain behavior of placenta; Neoplasm of uncertain behavior of ovary; Neoplasm of uncertain behavior of other and unspecified female genital organs |
| 256 | Ovarian dysfunction |
| 302.73 | Psychosexual dysfunction with inhibited female orgasm |
| 456.6 | Vulval varices |
| 614-629 | Inflammatory disease of female pelvic organs; Other disorders of female genital tract |
| 630-677 | Complications of pregnancy, childbirth and the puerperium |
| 752.0-752.4 | Anomalies of ovaries; Anomalies of fallopian tubes and broad ligaments; Doubling of uterus; Other anomalies of uterus; Anomalies of cervix, vagina, and external female genitalia |
| 792.3 | Nonspecific abnormal findings in amniotic fluid |
| 795.0 | Nonspecific abnormal papanicolaou smear of cervix |
| 867.4-867.5 | Injury to uterus |
| 878.4-878.7 | Open wound of vulva, including traumatic amputation; Open wound of vagina, including traumatic amputation |
| 939.1-939.2 | Foreign body in uterus, any part; Foreign body in vulva and vagina |
| 947.4 | Burn of vagina and uterus |
| V455 | Presence of contraceptive device |
